# Zinc Metalloproteins in Epigenetics and Their Crosstalk

**DOI:** 10.3390/life11030186

**Published:** 2021-02-26

**Authors:** Abdurrahman Pharmacy Yusuf, Murtala Bello Abubakar, Ibrahim Malami, Kasimu Ghandi Ibrahim, Bilyaminu Abubakar, Muhammad Bashir Bello, Naeem Qusty, Sara T. Elazab, Mustapha Umar Imam, Athanasios Alexiou, Gaber El-Saber Batiha

**Affiliations:** 1 Centre for Advanced Medical Research and Training, Usmanu Danfodiyo University, P.M.B. 2346 Sokoto, Nigeria; yusuf.abdurrahman@udusok.edu.ng (A.P.Y.); almalki.a@tu.edu.sa (I.M.); ghandi.kasimu@udusok.edu.ng (K.G.I.); abubakar.bilyaminu@udusok.edu (B.A.); mustapha.imam@udusok.edu.ng (M.U.I.); 2 Department of Physiology, Faculty of Basic Medical Sciences, College of Health Sciences, Usmanu Danfodiyo University, P.M.B. 2254 Sokoto, Nigeria; 3 Department of Pharmacognosy and Ethnopharmacy, Faculty of Pharmaceutical Sciences, Usmanu Danfodiyo University, P.M.B. 2346 Sokoto, Nigeria; 4 Department of Pharmacology and Toxicology, Faculty of Pharmaceutical Sciences, Usmanu Danfodiyo University, P.M.B. 2346 Sokoto, Nigeria; 5 Department of Veterinary Microbiology, Faculty of Veterinary Medicine, Usmanu Danfodiyo University, P.M.B. 2346 Sokoto, Nigeria; 6 Medical Laboratories Department, Faculty of Applied Medical Sciences, Umm Al-Qura University, Mecca 21955, Saudi Arabia; nfqusty@uqu.edu.sa; 7 Department of Pharmacology, Faculty of Veterinary Medicine, Mansoura University, Mansoura, Dakahlia 35516, Egypt; sarataha1@mans.edu.eg; 8 Department of Medical Biochemistry, Faculty of Basic Medical Sciences, College of Health Sciences, Usmanu Danfodiyo University, P.M.B. 2254 Sokoto, Nigeria; 9 Novel Global Community Educational Foundation, Hebersham, NSW 2770, Australia; 10 AFNP Med, Haidingergasse 29, 1030 Vienna, Austria; 11 Department of Pharmacology and Therapeutics, Faculty of Veterinary Medicine, Damanhour University, Damanhour, AlBeheira 22511, Egypt

**Keywords:** epigenetics, epigenome, zinc finger domain, zinc finger motif, zinc finger proteins, zinc metalloproteins

## Abstract

More than half a century ago, zinc was established as an essential micronutrient for normal human physiology. In silico data suggest that about 10% of the human proteome potentially binds zinc. Many proteins with zinc-binding domains (ZBDs) are involved in epigenetic modifications such as DNA methylation and histone modifications, which regulate transcription in physiological and pathological conditions. Zinc metalloproteins in epigenetics are mainly zinc metalloenzymes and zinc finger proteins (ZFPs), which are classified into writers, erasers, readers, editors, and feeders. Altogether, these classes of proteins engage in crosstalk that fundamentally maintains the epigenome’s modus operandi. Changes in the expression or function of these proteins induced by zinc deficiency or loss of function mutations in their ZBDs may lead to aberrant epigenetic reprogramming, which may worsen the risk of non-communicable chronic diseases. This review attempts to address zinc’s role and its proteins in natural epigenetic programming and artificial reprogramming and briefly discusses how the ZBDs in these proteins interact with the chromatin.

## 1. Introduction

Zinc is an omnipresent micronutrient essential for healthy prenatal and postnatal developments in humans and the growth and development of plants, animals, and microorganisms [1,2]. In silico data suggest that about 10% of the human proteome potentially binds zinc [3]. Zinc is present in all body tissues and fluids as a component of over 2000 proteins (including epigenetically active enzymes) [4,5,6,7]. A group of writers, erasers, and readers of epigenetic marks are zinc-dependent [8]. Some of these are enzymes such as histone deacetylases (HDACs) having the zinc itself incorporated in their active sites; thus, it directly partakes in the catalytic process [9]. Others are proteins containing zinc within a zinc-binding domain (ZBD). These domains are important in substrate recognition, self-regulation, integrity, crosstalk, and sometimes catalysis [10,11]. With intracellular zinc within the normal range, these proteins work together in a coordinated manner to shape the plastic epigenome. In contrast, fluctuations in zinc levels or its deficiency and loss of function mutations in the ZBDs of these proteins affect their expression and function and may lead to epigenetic perturbations. [12,13]. These aberrant epigenetic changes may increase the risk of non-communicable chronic diseases such as cancer, diabetes, and cardiovascular diseases with possible multigenerational or transgenerational consequences. Intracellular zinc homeostasis is under the tight regulation of two families of zinc transporters and the zinc-binding proteins metallothionines (MTs) [14,15]. In addition to their zinc homeostasis roles, zinc transporters are also emerging as important modulators of the epigenome [4].

The significance of zinc, its deficiency, its transporters, and some of its enzymes in epigenetics has been partly reviewed [4,8,16]. However, these reviews are not all-inclusive and did not cover other ZBD-containing proteins involved in epigenome programming and editing. Here, we attempt to provide a comprehensive overview of zinc metalloproteins’ roles in natural and synthetic epigenetics. We also try to explore how the ZBDs in these proteins interact with the chromatin. Of note, in the context of this article, the term “zinc metalloproteins” encompasses zinc finger proteins (ZFPs), zinc metalloenzymes, and enzymes requiring zinc activation for catalysis.

## 2. Molecular Bases of Epigenetic Modifications

Since the late 1980s, epigenetics has been one of the critical areas of research in molecular biology. From the late twentieth century to date, the meaning of the term “Epigenetics” has been evolving, and its exact definition remains elusive [17]. However, this review focuses on the most common definition (the molecular mechanism of transcription regulation) derived from the root words “epi+genetics”, which means in addition to or “on the top” of genetics. Based on this concept, it has been defined as the mitotically and meiotically heritable layer of chemical code beyond the deoxyribonucleic acid (DNA) sequence, which regulates the genome, leading to various transcriptional outcomes in different cell types [17,18,19]. The four basic mechanisms involved in epigenetic regulations include DNA methylation, histone post-translational modifications, remodeling of the chromatin architecture, and gene silencing associated with noncoding RNAs such as microRNA [20,21,22]. Discussing the detailed molecular mechanisms of these epigenetic events is indeed beyond the scope of this review. Therefore, here, we focus only on zinc’s role and that of its proteins in DNA methylation, histone modifications, chromatin remodeling, epigenome editing, and their crosstalk.

### 2.1. DNA Methylation

DNA methylation is the physical addition of a methyl (-CH_3_) group to a DNA sequence. The most common form of this modification in eukaryotes is adding a methyl group to the 5th carbon of cytosine in a DNA sequence to form 5′-methylcytosine (5′-mC) [23,24,25]. This modification predominantly occurs at cytosine-phospho-guanine dinucleotide-rich regions called CpG islands, which are mainly present around the promoter regions of genes and have been implicated in transcription regulation [26]. Moreover, DNA methylation occurs harmoniously and proportionately on both strands of the methylated DNA [27]. In most cases, cytosine methylation promotes gene silencing principally via the gene promoter’s CpG islands’ hypermethylation. Meanwhile, the promoter regions of transcriptionally active genes remain hypomethylated [26]. Thus, the methylation status of a gene could affect its expression level.

**Figure 1:**
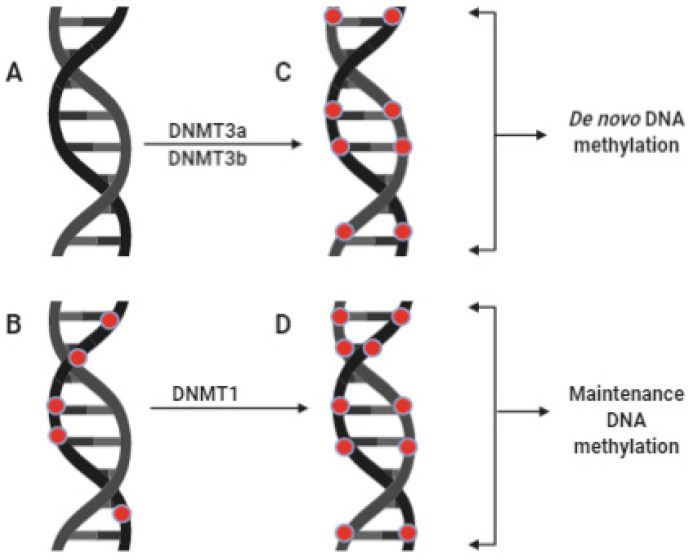
Comparison between de novo DNA methylation and maintenance DNA methylation. The figure compares de novo DNA methylation and maintenance DNA methylation. The two forms of DNA methylation catalyzed by DNMTs. (**A**) = unmethylated DNA; (**B**) = hemimethylated DNA; (**C)** and (**D**) = fully methylated DNA; DNMT3a/DNMT3b = DNA methyltransferases 3a/3b, both catalyze de novo DNA methylation (methylation of a newly formed double-stranded DNA during gametogenesis and embryogenesis). DNMT1 = DNA methyltransferase 1: It catalyzes replication-dependent maintenance of DNA methylation existing on hemimethylated DNA. Red dots stand for methyl groups. Source: Icons were obtained and customized in BioRender (biorender.com (accessed on 23 February 2021)).

In mammals, two sets of enzymes regulate the DNA methylation status of the genome. They include the zinc-dependent DNA methyltransferases (DNMTs), which facilitate 5′C methylation and its passive demethylation, and the iron-dependent members of the ten-eleven translocases (TETs), which catalyze the active demethylation of DNA coupled to base excision repair [23,28,29]. So far, DNMT1, DNMT3a, and DNMT3b are the three known DNMTs that directly add methyl groups from S-adenosyl methionine (SAM) to the human genome [30]. DNMT1 catalyzes the maintenance or restoration of DNA methylation patterns of a hemimethylated double-stranded DNA in somatic cells following replication; therefore, it is called maintenance DNMT (Figure 1) [31]. On the other hand, DNMT3a and DNMT3b establish new methylation tasks on a newly synthesized (unmethylated) double-stranded DNA in the germline and the embryo; they are named de novo DNMTs (Figure 1) [30]. These three enzymes are not mutually exclusive catalytically, as Ren et al. (2018) [31] have reported more evidence suggesting the involvement of each enzyme in de novo and maintenance DNA methylation.

### 2.2. Histone Post-Translational Modifications and Chromatin Remodeling

Histones are highly conserved basic proteins with a net positive charge. They have variable structures and amino acid composition that form the chromatin core. In eukaryotes, the chromatin core comprises a spherical octamer of four pairs of histone variants, namely H2A, H2B, H3, and H4, on which the DNA binds. Additionally, a fifth variant (H1) connects the DNA-bound octamers at regular intervals [32]. Each of the histone variants has an extended tail of amino acids at its N-terminus, which supports epigenetic modifications such as methylation, acetylation, phosphorylation, ubiquitination, ribosylation, citrullination, and SUMOylation [33,34,35,36]. Chromatin organization is achieved due to the tight winding of DNA around the histone octamers to form condensed structures called nucleosomes. Tightly packed nucleosomes form a higher-order structural organization called chromatin [22,37,38]. Based on the degree of packaging, chromatin can be heterochromatin or euchromatin. Heterochromatin is tightly packed and inaccessible to transcription factors, while euchromatin is loose and accessible [26].

Mechanistically, histone modifications regulate transcription in two ways: one is by facilitating the transition between heterochromatin and euchromatin (chromatin remodeling), and the other is by serving as scaffolds or binding sites for reader proteins, which in turn recruit other proteins that write or erase epigenetic marks [37,39,40]. It is conceivable that modifications that strengthen DNA–histone interactions to form tight chromatin usually lead to gene silencing, while those that disrupt this interaction promote gene expression [38]. Interestingly, histone modifications work in a coordinated manner. Different histone marks engage in unique crosstalk or interaction that maintains the chromatin’s highly dynamic state. This interaction will be discussed further under this review’s subsequent headings, describing zinc’s role in the various forms of histone modifications.

## 3. Molecular Bases of Epigenome Regulation Associated with Zinc Metalloproteins

Some of the epigenetically active enzymes identified so far are zinc metalloenzymes. Zinc is essential for the catalysis and autoregulation of enzymes such as DNMTs, histone methyltransferases/methylases (HMTs), histone demethylases (HDMs), histone acetyltransferases/acetylases (HATs), histone deacetylases (HDACs), histone E3-ubiquitin ligases (EUBLs), and histone deubiquitinating module (DUBm) complexes [4,41]. Interestingly, zinc is not only required for the integrity, catalysis, and self-regulation of these enzymes but also in recognition of their substrates and their recruitment to binding sites by other zinc-binding proteins [31,42,43,44]. Furthermore, the methionine synthase and betaine–homocysteine methyltransferase essential in DNA and histone methylation are zinc-dependent [45]. Additionally, zinc finger proteins (ZFPs) are also involved in epigenetic regulations, especially in epigenome editing [46]. ZFPs are the largest group of transcription factors with diverse structures that commonly have a zinc finger domain (ZFD) housing structural arrays of amino acids coordinated by one or more zinc atoms called zinc finger motifs (ZFMs) [10,47].

### 3.1. Role of Zinc in DNA Methylation

Each of the three DNMTs is composed of complex multi-functional domains categorized into a C-terminal catalytic domain and an N-terminal regulatory domain [31]. DNMT1 contains approximately 1620 amino acid residues [48]. About 78 of the total amino acids (about 4.83%), which comprises residues 621–698, form a ZBD called the CXXC domain (C for Cysteine and X for any amino acid) [49]. The domain is part of the N-terminal regulatory region, which interacts with the unmethylated CpG islands of a hemimethylated DNA to facilitate the self-inhibition of DNMT1 through an autoinhibitory linker. In this way, the enzyme is prevented from de novo DNA methylation [31,50,51]. Furthermore, the interaction between the ZBD and the catalytic domain of DNMT1 is essential for the enzyme’s allosteric activation [4]. Thus, the ZBD forms part of the N-terminal regulatory domain that controls the enzyme’s catalytic activity. Additionally, the ability of DNMT1 to recognize hemimethylated DNA is dependent on its interaction with a ZFP called the ubiquitin-like protein 1, containing plant homeodomain (PHD) and a really interesting new gene (RING) finger domains (UHRF1). This protein senses DNMT1 and reads methylation patterns on a hemimethylated DNA with the aid of its PHD and SRA (SET and RING associated) domains [43]. Thus, it recognizes the methylation marks on the DNA and directs the enzyme to those marked regions [44,52]. Similarly, the N-terminal regulatory domains of both DNMT3a and DNMT3b harbor a cysteine-rich complex multi-subunit domain called the “alpha thalassemia mental retardation x-linked DNA methyltransferase3 DNA methyltransferase3L” related domain abbreviated as *ATRX-DNMT3-DNMT3L* or simply ADD domain. The domain encloses two ZFDs, namely a Guanine-Alanine-Thymine-Alanine-like (GATA-like) and a PHD together with a C-terminal alpha-helix [42,53]. ADD domain facilitates the autoinhibition and allosteric activation of both enzymes [31]. In addition to DNMT3a and b, DNMT3-like protein (DNMT3L) is another member of the DNMT3 family identified in humans [54]. This protein also contains the ADD domain but lacks the catalytic domain and helps in the allosteric regulation of both DNMT3a and DNMT3b [27,42]. In 2001, Bourc’his and co-researchers reported that some offspring of a homozygous knockout (DNMT3L-/-) model of female mice died before midgestation [55]. According to these researchers, the dead fetuses had hypomethylated maternally imprinted genes. This finding suggests the role of DNM3L in the de novo methylation of maternally imprinted regions of the DNA. A year later, Chedin et al. (2002) [54] co-expressed DNMT3L with DNMT3a in a human cell line. Their intervention enhanced the de novo methylation activity of DNMT3a at the targeted DNA sequences irrespective of which sequence is involved, but with little or no effect on DNMT3b. However, studies reviewed by Suetake et al. (2004) [56] have reported similar results for DNMT3b. These observations entail that DNMT3L has a stimulatory role on both DNMT3a and DNMT3b. Mechanistically, the stimulatory role of DNMT3L on DNMT3a/b may not involve their recruitment to the targeted regions. Instead, it may depend on the allosteric interaction between the C-terminal half of DNMT3L, and these enzymes’ catalytic domains [56]. Moreover, DNMT3L could bind to DNMT3a/b but not the DNA itself [56], further emphasizing the lack of the protein’s catalytic domain. Furthermore, a more recent study indicated that DNMT3L exerts its regulatory function through its PHD-like ZFD (the ADD domain) [57]. In a nutshell, these findings imply that the ZFD (ADD) in the DNMT3 family dictates the enzymes’ catalytic domains.

Emerging evidence suggests a strong correlation between cellular zinc levels and the expression and activities of DNMTs in different experimental models. For instance, studies have shown a significant increase in the protein expression levels and activities of DNMT1 and DNMT3A in zinc-deficient human esophageal cancer (EC) cell lines compared to similar cell lines without zinc deficiency [58,59]. Consequently, zinc deficiency improved these cells’ radiosensitivity by enhancing the hypermethylation of the microRNA 193b gene promoter via the upregulation of DNMTs. MiR-193b induces radioresistance in EC cells by arresting their cell cycle through the downregulation of Cyclin D1 mRNA [58]. One possible mechanism by which zinc deficiency could upregulate DNMT1 activity is by distorting the N-terminal CXXC domain’s integrity due to lack of zinc, which may result in loss of its autoinhibitory function.

Moreover, in another study on hypozincemia-induced cognitive dysfunction in rats, zinc deficiency led to the upregulation of DNMT1 transcription in their hippocampus and subsequent hypermethylation of the brain-derived neurotrophic factor (BDNF) gene and its downregulation [60]. BDNF is highly expressed in the mammalian brain’s hippocampus and is one of the critical regulators of learning and memory [61,62]. In contrast, no significant change in DNMT3B gene expression was reported in the study, while DNMT3A expression was downregulated [60]. Furthermore, a more recent study on the effect of zinc supplementation on offspring’s cognitive function in rats reported a significant downregulation of DNMT1 and BDNF as well as the upregulation of DNMT3A protein levels in the hippocampus of the F1 neonates of zinc-deficient dams; however, all three proteins were downregulated in the lactating offspring of these rats [63]. According to the researchers, there was no significant hypermethylation of the BDNF gene in the offspring throughout the experiment. Its downregulation at the developmental stage correlates with the neonatal upregulation of DNMT3A, which may be due to the deformation of its regulatory ZBD (the ADD domain).

Additionally, this observation further illustrates the enzyme’s de novo methyltransferase activity described earlier. However, there were no significant changes in the protein expression levels of DNMTs and BDNF following post-weaning zinc supplementation, although DNMT3A and BDNF showed an increasing trend [63]. This finding implies that zinc supplementation in offspring could restore the expression of DNMTs and DNA methylation changes in some genes, which an early life zinc deficiency might have perturbed in parents. Thus, it is conceivable from these observations that zinc deficiency affects the DNA methylation statuses of genes through different pathways involving DNMTs in vivo. It is also noteworthy that hypozincemia-induced perturbations in the DNA methylation status depend on the cell’s physiological or pathological state.

#### Role of Zinc in Folate-Mediated One-Carbon Metabolism

Zinc indirectly affects DNA and histone methylation through one-carbon metabolism, which is a term used to describe the relationship between folate and methionine metabolic pathways [64]. One carbon unit (such as methyl and formyl groups) is transferred to folic acid from amino acids and then redistributed to other molecules through SAM to facilitate methylation reactions, including DNA and histone methylations [65].

**Figure 2:**
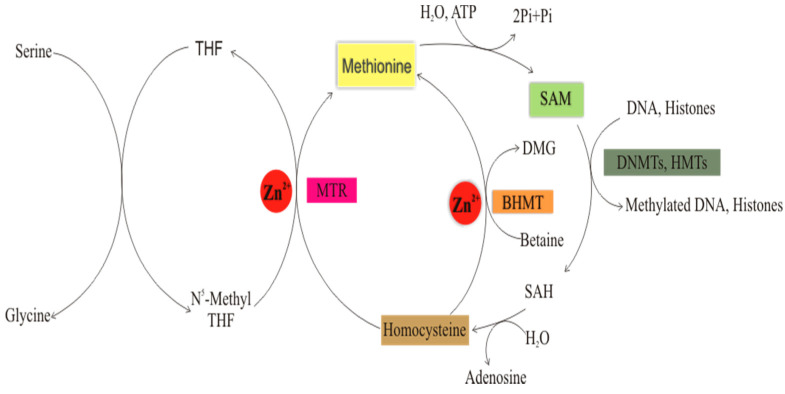
Role of zinc in DNA and histone methylation through the folate-mediated one-carbon metabolism. This figure summarizes the pathways linking one-carbon metabolism with DNA and histone methylation. Zinc-dependent enzymes and key intermediates are highlighted in different colors. BHMT (**orange**) = Betaine homocysteine methyltransferase: A zinc-dependent enzyme, which catalyzes the alternate reaction of methionine synthesis; DMG = Dimethylglycine; DNMTS (**dark green**) = DNA methyltransferases: Catalyze DNA methylation and have zinc-binding domains that regulate their activities; HMTs (**dark green**) = histone methyltransferases: Catalyze histone, lysine, and arginine methylation; MTR (**pink**) = Methionine synthase: It is zinc-dependent and catalyzes the direct synthesis of methionine (the direct precursor of SAM) from homocysteine and N^5^-methyl THF; SAH = S-Adenosylhomocysteine; SAM (**light green**) = S-Adenosylmethionine: The activated methyl donor for methylation reactions; THF = Tetrahydrofolate: The active form of folate that supplies methyl groups to homocysteine to form methionine. Zn2+ (**red**) = Zinc ion. Source: Image was created in Corel Draw X3 Graphics Suite (Pixmantec Rawshooter essentials 2005. Version 1.1.3).

The methyl groups added to DNA and histones usually come from amino acids, such as serine and betaine/trimethylglycine (obtained from choline). Serine donates a 1C unit to form glycine and 5, 10-methylenetetrahydrofolate. Then, the latter is reduced to 5-methyltetrahydrofolate by methylenetetrahydrofolate reductase (MHTR). Then, the activated folate donates its methyl group to homocysteine to form methionine, and thus, THF is regenerated for another cycle. This reaction is catalyzed by 5-methyltetrahydrofolate-homocysteine methyltransferase (MTR), which is also known as methionine synthase [65,66]. MTR is dependent on zinc and biotin [16]. In the active site of MTR, the zinc ion is coordinated by three cysteine residues, namely C217, C272, and C273, in a trigonal bipyramidal structure [67], which is critical for the catalytic activity of this enzyme. Following synthesis, methionine reacts with ATP to generate SAM, which facilitates DNA, RNA, and histone methylation, among other methylation reactions, and homocysteine is regenerated for another cycle. A study has shown that MTR gene expression was significantly downregulated in the liver and kidney of zinc-deficient rats compared to control. These changes could not be reversed by zinc supplementation [68]. The study also reported elevated levels of homocysteine (an immediate substrate of MTR) in the serum of zinc-deficient rats, further indicating the enzyme’s downregulation. One possible consequence of this finding is the depletion of SAM (a cofactor of DNMTs and HMTs), thus affecting DNA and histone methylation (Figure 2).

Alternatively, methionine is generated from homocysteine and betaine in another reaction catalyzed by betaine–homocysteine methyltransferase (BHMT) [64,65,69]. This enzyme also requires zinc for its activity [16] (Figure 2). In the active site of BHMT, the zinc atom is coordinated by the thiol groups of three cysteine residues, namely C217, C299, and C300 tetrahedrally, in addition to a fourth, which is coordinated by the OH of tyrosine (Y77). This coordination is also critical for the enzyme’s catalytic activity [70]. In a study involving mice initially fed with a high-fat diet and later treated with either a zinc diet (containing 30 g zinc) or a no-added zinc diet, there was a significant downregulation of the BHMT gene and protein expression, lower methionine levels, and reduced homocysteine clearance in the liver of zinc-deficient mice; the mRNA and protein levels of Specificity protein 1/Sp1 (a ZF transcription factor that regulates the transsulfuration pathway of methionine metabolism) were also suppressed in the same group of mice [71]. Notably, these observations reaffirm the vital role of zinc in methionine and SAM synthesis as well as homocysteine clearance, which is mechanistically due to its ability to regulate the expression and function of MTR, BHMT, Sp1, and perhaps other unidentified zinc metalloproteins involved in the various pathways linking homocysteine to SAM. Thus, the findings explain zinc’s role in maintaining DNA and histone methylation facilitated by SAM, which is a product of one carbon metabolism and a cofactor of DNMTs and HMTs.

### 3.2. Role of Zinc in Histone Methylation

Histone methylation is the addition of one, two, or three methyl groups on the ε-nitrogen of lysine or the guanidino nitrogen of arginine residues of mainly H3 and H4 tails, which is catalyzed by HMTs [72,73]. Histone arginine methylation is catalyzed by protein arginine methyltransferases (PRMTs). In contrast, histone lysine methylation is catalyzed by lysine-specific histone methyltransferases containing or not containing the evolutionarily conserved SET domain (suppressor of variegation, enhancer of zeste, and trithorax) [72,74]. SET is a catalytic domain initially discovered in the expression product of some genes responsible for heterochromatin formation and the white eye phenotype in Drosophila melanogaster [75]. In some SET domain-containing HMTs, the domain harbors either post-SET only or pre-SET and post-SET cysteine-rich ZFDs. These domains interact with the SET domain to maintain its integrity and regulate its catalytic activity [73,74]. For instance, the two yeast HMTs, cryptic loci regulator 4 and histone-lysine N-methyltransferase dim-5 (Clr4 and Dim-5) are SET domain-containing HMTs, and both have pre-SET and post-SET ZFDs flanking their SET domain [76,77]. Clr4 is a reader and a writer of histone 3 lysine 9 (H3K9) methylation mark and facilitates chromatin condensation in Schizosaccharomyces pombe [76]. On the other hand, Dim-5 methylates H3 at K9 and facilitates DNA methylation in Neurospora crassa [78]. The pre-SET ZFD of both enzymes exerts a structural function and encloses three zinc ions in trigonal coordination with nine cysteine residues. Each zinc ion bonds to four cysteine residues tetrahedrally such that three are individually attached while it shares the fourth one with the neighboring zinc atom [76,79]. The post-SET domain in both proteins appears to be a cysteine-rich ZFD enclosing a zinc ion in tetrahedral coordination. It seems to be involved in SAM binding, although its exact function remains elusive [74,79]. Histone lysine methyltransferases are very diverse in eukaryotes, including humans. About seven families and a few orphan members have been identified in humans. SUV39H1 and SUV39H2 are examples of human homologs of the yeast Clr4 harboring the pre-SET and post-SET ZFMs [79,80]. SET domain-containing HMTs catalyzes the methylation of H3, mainly at K9 and K4 [45,76]. Interestingly, histone lysine methylation does not disrupt the lysine residues positive charge and has fewer effects on DNA–histone interaction. However, unlike DNA methylation that often leads to gene silencing, histone lysine methylation activates or represses genes either by providing a binding site for methyl reader proteins, which in turn recruit the transcription machinery to the targeted genes or by remodeling the chromatin architecture, thus affecting the ability of transcriptional complexes to access DNA [23,52,81]. Generally, the methylation of H3 at K4, K36, and K79 leads to gene activation, whereas methylation at K9 and K27 of H3, as well as K20 of H4, altogether leads to gene repression [45].

### 3.3. Role of Zinc in Histone Demethylation

Histone demethylation is the reverse of histone methylation catalyzed by HDMs. It involves the removal of methyl groups from the lysine and arginine residues of histone tails. Histone demethylases are of two types: lysine demethylases (KDMs) and peptidyl-arginine demethylases (PADs) [82]. KDMs are the class of histone demethylases with ZBDs. Based on their catalytic mechanisms, they are subdivided into two groups of six families: a flavin adenine dinucleotide-dependent amine oxidases (AOF/KDM1) and an iron (ii) and alpha-ketoglutarate-dependent Jumonji-containing (JmjC) dioxygenases (KDMs 2-6) [4,83]. Two members of the KDM1 family, lysine-specific demethylases 1 and 2 (LSD1 and LSD 2), have been identified [80]. LSD2, also known as KDM1B or AOF1, has approximately 822 amino acids, some of which form an N-terminal ZFD with C4H2C2-type and CW-type classes of ZFMs, and specifically demethylates H3K4me1 and H3K4me2 marks [84]. The N-terminal ZFD (residues 50–264) facilitates substrate specificity and maintains the enzyme’s active conformation [85]. Mutations in the genes transcribing the ZF components of this domain induce conformational changes in the amine oxidase domain, with subsequent loss of the demethylase activity [86]. This observation suggests its role in the catalytic mechanism of the enzyme. The second family of lysine demethylases encompasses more than 30 proteins, all of which contain the JmjC domain [82]. A typical example of a KDM in this family is KDM2B, also known as *JHDM1B/FBXL10/NDY1*, which demethylates H3K4me3 and H3K36me2. In addition to the JmjC domain, the enzyme contains two other zinc finger domains: the CXXC and the PHD domains, together with an F-box domain. The JmjC domain erases the H3K36me2 mark, the CXXC is for unmethylated DNA binding and recruitment of transcription factors, and the PHD serves as a histone modificatison reader domain [83]. A study has reported the enrichment of the H3K4me3 mark in the thymus of hematopoietic cell-specific knockout mice model of the CXXC domain of KDM2B [87]. This observation supports the earlier mentioned role of this domain in the demethylation of the H3K4me3 mark.

### 3.4. Role of Zinc in Histone Acetylation

Histone acetylation is the transfer of a negatively charged two-carbon unit, the acetyl group (CH_3_CO^−^), from acetyl-CoA to specific N-terminal lysine residues of histone tails [88]. Being negatively charged, the acetyl group neutralizes the positive charge on the acetylated lysine residues and hence reduces the overall positive charge on the histone protein. Consequently, this leads to the disruption of DNA–histone interactions (electrostatic interactions between negatively charged DNA and the positively charged histones), making the DNA more accessible to the transcription machinery. Therefore, histone acetylation favors euchromatin formation and activates gene expression [4,88,89,90]. Histone acetylation is catalyzed by HATs, some of which are zinc-dependent. Several families of HATs have been identified in eukaryotes, including humans [91]. Examples of ZBD-containing HATs are the members of the MYST family, which is an acronym derived from the names of two human genes and two yeast genes: human monocytic leukemia ZFP (MOZ), yeast bf2, also known as ySas3 or KAT6 (histone lysine acetyltransferase 6), yeast Sas2 (KAT8), and human Tip60 (KAT5). The defining feature of this class of HATs is the N-terminal MYST domain, which has an intrinsic HAT activity [92]. Some MYST family members contain zinc within a C2HC-type ZFM and a PHD-linked ZFD, which helps in the identification of and interaction with substrates [91,93]. Five members of the MYST family have been identified in humans: two important ones are MOZ and its paralog MORF (MOZ Related Factor), which are also known as KAT6A and KAT6B (previously MYST3 and MYST4), respectively; both catalyze the acetylation of H3 at K9 and K14 [94]. Similarly, the N-terminals of both enzymes enclose two tandem PHD ZFs that enable H3 recognition and binding and regulate the HAT domain [93,94].

### 3.5. Role of Zinc in Histone Deacetylation

Histone deacetylation is the hydrolytic removal of an acetyl group from N-acetyl lysine residues on histone tails, which are catalyzed by HDACs. A total of eighteen HDACs hydrolyze the amide bond of N-acetyl lysine residues of histone tails in mammals, including humans [95]. These enzymes are of four categories or classes, which require either zinc or NAD+ for catalysis. Class I (HDACs 1-3, 8), class II (IIa: HDACs 4, 5, 7, 9 and IIb: HDACs 6, 10) and class IV (HDAC 11) are zinc-dependent, while class III (sirtuins 1-7) depend on NAD+ [96,97,98,99]. The zinc ion in the active sites of zinc-dependent HDACs coordinates an aspartate-histidine (D-H) dyad and a tyrosine (Y) residue (except in class IIa HDACs, in which the Y residue has been replaced by another H). The coordination maintains the appropriate positioning of the functional groups required for the catalytic process [9,97,100]. In this catalytic mechanism, the zinc ion launches an initial nucleophilic attack on the water molecule (an essential step in the catalytic mechanism common to numerous zinc metalloenzymes, including carboxypeptidase A and carbonic anhydrase). Then, the zinc ion induces a polarity on the carbonyl oxygen of N-acetyl lysine on the histone tail and thus facilitates a broad base nucleophilic attack (on the carbonyl carbon) by the zinc-bound water molecule [9,100,101]. In addition to the central zinc atom typical to all the eleven zinc-dependent HDACs, class IIa HDACs also enclose another zinc atom within a unique and highly conserved (among members of this class IIa) CCHC-type ZFM found adjacent to the entrance of their active sites [102]. Although this motif’s exact function is not clear, it may be critical for maintaining these enzymes’ catalytic activity and stability and may serve as a potential allosteric site to develop their inhibitors. HDAC inhibitors have been employed in the management of diabetes mellitus (DM) [103,104,105] and cancer [100,106,107]. Interestingly, some of these drugs’ inhibitory mechanism is based on their ability to chelate the zinc ion in their active sites [9].

### 3.6. Role of Zinc in Histone Ubiquitination

Ubiquitination is the covalent attachment of one or a chain of ubiquitin molecules. It is one of the diverse post-translational medications of proteins, including histones [108]. Histone ubiquitination (mostly monoubiquitination) occurs predominantly at specific lysine residues of H2A and H2B. It is involved in transcription regulation and DNA repair [42]. This modification regulates transcription by three primary mechanisms: (1) direct remodeling of the chromatin architecture to increase or decrease DNA accessibility to transcriptional complexes, (2) recruitment of proteins that facilitate chromatin remodeling, and (3) as a prerequisite for other histone modifications such as methylation and acetylation [109]. Generally, protein ubiquitination occurs in three stages: activation, conjugation, and ligation catalyzed by ubiquitin-activating enzymes (E1), ubiquitin-conjugating enzymes (E2), and ubiquitin ligases (E3) [110].

The zinc-dependent class of E3 ubiquitin ligases (EUBLs) are the RING domain-containing proteins that catalyze the monoubiquitination of H2A at K13, K15, K119, K127, and K129 as well as H2B at K34, K120, and K125, respectively [41]. The RING domain harbors two zinc atoms in tetrahedral coordination with a histidine ring and seven cysteine side chains. This arrangement creates a C3HC4-type ZFM with 40–60 amino acid residues arranged in the sequence, Cys-X2-Cys-X [9-39]-Cys-X[1-3]-His-X[2-3]-Cys-X2-Cys-X[4-48]-Cys-X2-Cys, where “X” represents any amino acid [111]. Histone ubiquitination marks are associated with transcription silencing, activation, and DNA repair or participate in crosstalk that regulates other histone marks, such as histone methylation, which is essential in maintaining the dynamic nature of the chromatin [41,112].

In humans, H2A monoubiquitination is catalyzed by three enzymes or enzyme complexes: (1) RING finger 168 (RNF168), which adds a unit of ubiquitin on H2A at K13 or K15 and promotes the non-homologous end-joining pathway in the DNA damage repair response; (2) members of the polycomb repressor complex 1 (RING1A, RING1B and RNF51), which ubiquitinate H2A at K119 and lead to transcriptional repression; (3) the BRCA1/BARD1 complex (BReast CAncer type-1 susceptibility protein/BRCA1-Associated RING Domain protein 1) ubiquitinates H2A at K127 K129 and promotes the homologous recombination pathway in the DNA damage repair response [112]. On the other hand, several RING finger families of enzymes and enzyme complexes that catalyze H2B monoubiquitination have been identified in humans. The RNF20–RNF40 complex, which mainly catalyzes H2BK120ub1, appears to be the predominant writer of H2Bub1 marks [41,112]. Another important class is the MOF–MSL (MOZ-related Factor–Male Specific Lethal homolog) complex comprising MOF, MSL1, MSL2, and MSL3. The MSL1/2 component catalyzes the monoubiquitination of H2B at K34, while the MOF component (a member of the MYST family of HATs discussed earlier) catalyzes H4K16 acetylation [113]. Of note, H2Bub1 at K120 and K34 has been linked to transcriptional upregulation. In contrast, H2Aub1 at K34 and K120 and H4K31ub1 are collectively involved in crosstalk that facilitates H3K4 and H3K79 methylation [41].

### 3.7. Role of Zinc in Histone Deubiquitination

Histone deubiquitination is the removal of the ubiquitin moieties attached to lysine residues of histone tails by hydrolysis. H2A/B deubiquitination is catalyzed by the *Spt-Ada-Gcn5-Acetyltransferase* (SAGA) coactivator complex, which is a multi-subunit complex with both histone acetylase and deubiquitinase activity that is structurally and functionally conserved from yeast to humans [114]. In both yeast and humans, the SAGA complex is organized into a functional unit called the deubiquitinating module (DUBm), comprising of four functional subunits: three of them form an N-terminal regulatory region, and the fourth one creates the catalytic domain [115]. The four components in the yeast DUBm are the ubiquitin-specific protease 8 (Ubp8), which is the catalytic domain, and the three N-terminal subunits, namely the SAGA-associated factor 11 (Sgf11), the transcription and mRNA export factor (Sus1), and the SAGA-associated factor 73 (Sgf73). These units altogether serve as a scaffold that maintains the catalytic domain’s active form [115]. Of note, Sgf11 and Sgf73 are ZFDs required for the DUBm function and stability, respectively [116]. In humans, the ubiquitin-specific protease 22 (USP22) is the ZFD-containing component of the human SAGA complex that deubiquitinates both H2A and H2B in vitro [114]. Moreover, similar to the yeast complex, this module also contains hATXN7 (human ataxin 7), hATXN7L3 (human ataxin 7 like 3), and hENY2 (human enhancer of yellow 2 transcription factor homolog, which are the ZBD-containing protein orthologs of the yeast Sgf73, Sgf11, and Sus1, respectively. The units interact to allosterically regulate its activity [114].

### 3.8. Zinc Finger Proteins That Read Epigenetic Modifications

Apart from enzymes, a plethora of proteins containing ZBDs (collectively referred to as transcription factors) is also involved in epigenetics. These proteins can read/recognize and bind to methylation patterns on the CpG islands of DNA or the various modifications on the N-terminal lysine/arginine residues of histone tails and then recruit other proteins that alter chromatin conformation, leading to varying transcriptional consequences [10,11,117].

#### 3.8.1. DNA Methylation Readers and Their Binding Mechanisms

**Figure 3:**
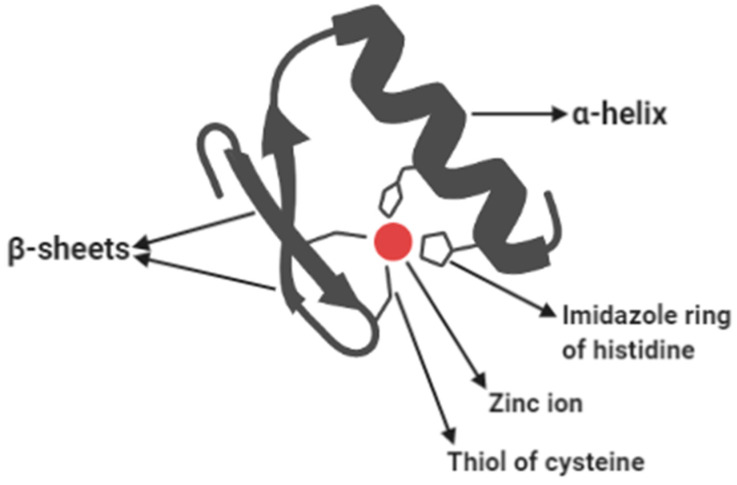
Labeled cartoon representation of a Cys2His2 zinc finger motif. A C2H2 zinc finger is an essential structural component of fully methylated DNA reader proteins with methyl binding domains. The red dot represents the zinc atom; the spiral structure denotes the alpha-helix; the two antiparallel arrows are the beta-sheets. The two five-membered rings extending from the alpha helix are the imidazole rings of histidine; the two curves extending from the beta-sheets and pointing toward the red dot are the thiols of the two cysteine residues. Source: Icon was obtained and customized in BioRender (biorender.com (accessed on 23 February 2021)).

Based on the commonalities in their methyl binding domains (MBDs) and the binding mechanism, methyl binding proteins (MBPs) are of two types: readers of double (fully) methylated DNA and readers of hemimethylated DNA. The first group (readers of double-methylated DNA) comprises the best-characterized families of MBPs [10]. The most typical feature uniting these diverse families of transcription factors is the presence of two or more Cys2His2 (C2H2) ZFMs. Each ZFM has approximately 30 amino acid residues arranged in a simple structure: a central zinc atom coordinating two histidine rings protruding from the α-helix and two cysteine side chains extending from the β-sheets (Figure 3). Then, these motifs form binding domains harboring a set of two, three, or more zinc fingers arranged in tandem repeats. Examples include the Broad-complex, Tramtrack, and Bric-a-brac, which are also known as POxvirus and Zinc finger (BTB/POZ) domains containing families such as Kaiso (ZBTB33), ZBTB4, and ZBTB38 [10,117,118].

More members of ZF MBPs reviewed by Hodges et al. (2020) [10] include zinc finger protein 57 (ZFP57), a member of the Krüpple-associated box (KRAB) domain family; Krüppel-like factor 4 (KLF4), a member of the Krüppel-like factor family, Wilm’s Tumor Gene 1 (WT1); Early Growth Response Protein 1 (EGR1); and CCCTC-binding factor (CTCF), which is also known as 11-ZFP.

These proteins’ binding mechanisms are complex and transcription factor-dependent due to variable structures and amino acid composition. However, they generally present a unique interaction model. Here, the arginine residue that precedes the first histidine ring in the C2H2 ZFM forms a hydrogen bond with the 3′-G ring and a Van der Waals interaction with the 5′-mC of the CpG dinucleotide. This interaction creates a 5-methylCytosine/Thymine-Arginine-Guanine triad typical of fully methylated DNA readers [119]. Additionally, a conserved glutamate residue (among the readers of double methylated DNA) also interacts with the 5′-mC via an OHN-type and an OHC-type hydrogen bonds [10]. This mechanism has been extensively reviewed recently [117,120]. Moreover, a conserved lysine residue in ZBTB38 and other ZFPs (whose ZFM sequences were aligned) substitutes the arginine in methylated DNA binding [117]. Thus, this finding demonstrates another possible mechanism for CpG methylation recognition by ZFPs.

On the other hand, the hemimethylated DNA readers are mainly the SRA domain-containing families of MBPs [121]. UHRF1 and UHRF2 are the members of this family identified in humans. In addition to the SRA domain, both contain a ubiquitin-like (UBL), a tandem Tudor, a PHD, and a RING domain. Studies have shown that the ability of UHRF1 to recruit DNMT1 to the replication foci depends on the interaction between its SRA domain and hemimethylated DNA [31,43,44]. UHRF2 is also known to read the 5-hydroxymethyl cytosine (5 hmC) mark on DNA through its SRA domain and recruit DNMT1 to replicate foci [122]. 5 hmC is an oxidation product of 5 mC generated by the members of the TET family of proteins in the mammalian DNA demethylation pathway; it is found at the proximity of transcription factor binding sites and is also involved in the regulation of transcription [123,124].

#### 3.8.2. Histone Modifications Readers and Their Binding Mechanisms

Readers of histone modifications are numerous, very diverse, and histone mark specific. The best characterized ZF readers include bromodomain, chromodomain, and PHD readers. They recognize modified and unmodified lysine and arginine residues [125]. The chromodomain and bromodomain proteins recognize methylated lysine and acetylated lysine, respectively. In contrast, the PHD readers acknowledge both modified and unmodified lysine and arginine residues. Thus, PHD proteins are considered versatile readers of histone modifications [40].

The predominant readers of lysine methylation are the chromodomain containing members of chromatin modifiers (collectively referred to as the Royal family), which recognize methylated lysine mainly at H3 and participate in chromatin remodeling. A critical member of this family is the heterochromatin protein 1 (HP1). It binds to H3 methylations primarily at K9 and, to some extend K27, and it facilitates heterochromatin condensation [126]. Another important example of a methylated lysine reader is the Bromodomain PHD finger Transcription Factor (BPTF). This protein is a PHD containing a bromodomain reader considered the largest subunit of the ATP-dependent chromatin remodeling complex called the nucleosome remodeling factor (NURF) in humans. This protein has a preference for H3K4me2/me3 (the marks associated with the transcription start site of active genes) and thus remodels the chromatin state of those genes to enable their expression [127].

Due to their diversity, the binding mechanisms of methyl lysine readers differ from one reader to another. However, most of these proteins form an aromatic pocket with the side chains of tryptophan and tyrosine residues, which help them recognize and bind to various histone methylation marks. On the other hand, readers of unmodified lysine such as the PHD do not form an aromatic pocket; instead, they form hydrogen bonds between the ε-nitrogen of the lysine residue and the hydroxyl hydrogen of either aspartate or a glutamate residue in the PHD domain [125]. Table 1 summarizes the significant ZBDs discussed in this review; their functions and examples of human proteins containing these domains are given.

### 3.9. Role of Zinc in Epigenome Editing

Due to their ability to recognize epigenetic marks, zinc finger proteins (especially the Cys2His2 class of ZFs) are also involved in synthetic epigenetics. It is a popular concept known as epigenome editing [128]. This synthetic form of epigenetic modification entails writing or erasing epigenetic marks on the DNA or histones via the recruitment of the natural catalytic activity of the chromatin-modifying enzymes [46]. Two basic techniques have been employed to achieve this purpose. One of them involves using artificially customized epigenetic mark readers (the Cys2His2 ZFs) to recruit these catalytic domains to the predetermined targeted regions on the DNA and histones [20]. The ZFs are coupled to the chromatin-modifying enzymes, and the resultant machinery is employed to add or erase epigenetic marks.

The abbreviations used in the table are defined hereunder, and the respective letters in each acronym are capitalized. ADD—Alpha thalassemia mental retardation x-linked DNA methyltransferase3 DNA methyltransferase3L; BTB/POZ—Broad-complex, Tramtrack, and Bric-a-brac, also known as POxvirus and Zinc finger; BPTF—Bromodomain PHD finger Transcription Factor; CXXC—C for Cysteine and X for any amino acid; DNMT1—DNA methyltransferase1; DNMT3a/3b/3L—DNA methyltransferase3a/3b/3L; HAT—Histone acetyltransferase; H3K36me2—Histone 3 lysine 36 dimethyl; H3K4—Histone 3 lysine4; H3K9/K14—Histone 3 lysine 9/lysine14; JmjC—Jumonji-containing; KDM2B—lysine demethylase 2B; KRAB—KRüpple-Associated Box; MORF—MOZ-Related Factor; MOZ—human MOnocytic leukemia Zinc finger protein; MYST—MOZ, Ybf2 (Sas3), Sas2, and Tip60; PHD—Plant HomeoDomain; RING—Really Interesting New Gene; RNF168—Ring Finger168; PRC1—Polycomb Repressor Complex1; RNF20–RNF40—Ring Finger20–Ring Finger40; SET—Suppressor of variegation 3-9, Enhancer-of-zeste and Trithorax; SET1A—Suppressor of variegation 3-9, Enhancer-of-zeste and Trithorax 1A; SET1B—Suppressor of variegation 3-9, Enhancer-of-zeste and Trithorax 1B; SRA—SET and RING finger Associated domain; UHRF1—Ubiquitin-like, containing PHD and RING finger domains, 1; UHRF2—Ubiquitin-like, containing PHD and RING finger domains, 2; ZFP57—Zinc finger protein 57.

#### Role of Zinc in DNA Methylation Editing

**Figure 4:**
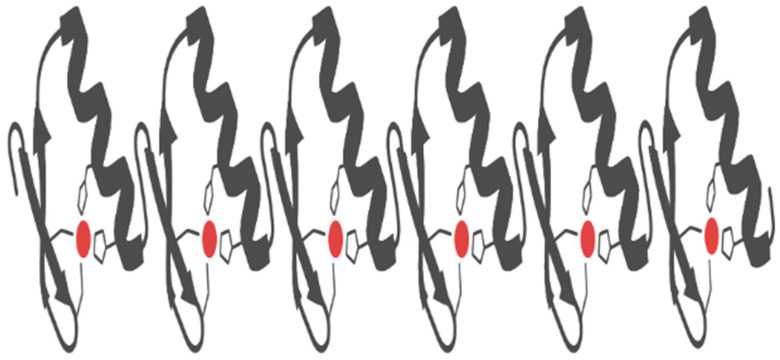
Cartoon representation of the zinc finger (ZF) array. A customized sequential array of six Cys2His2 ZFs: each of the six units containing a red dot surrounded by a coiled strand and two antiparallel arrows represent a Cys2His2 ZF. The red dot is the zinc ion coordinated by the side chains of two cysteines and two histidine residues. ZF arrays are employed in synthetic epigenetics to edit (add or delete) epigenetic marks at predetermined DNA or histone regions. They are usually coupled with writers or erasers to achieve this purpose. Source: Icons were obtained and customized in BioRender (biorender.com (accessed on 23 February 2021)).

DNMTs coupled to zinc fingers are used to introduce or delete DNA methylations at specific predetermined targeted regions. This technique has been used to alter the DNA methylation statuses of genes, affecting gene expression. For example, a customized array of seven ZFs coupled to the catalytic domain of DNMT3a was employed to induce DNA methylation on the p16 promoter of immunodeficient mice. Consequently, this led to increased metastasis and cancer cells’ proliferation due to the p16 gene suppression [129]. P16 is a tumor-suppressor protein that was found to be associated with improved prognosis and early survival of patients with oropharyngeal cancer in China [130]. In ZF arrays, each unit of a ZF recognizes three nucleotide bases. Hence, the customized collection of zinc fingers used in chromatin recognition can bind to about nine to 18 DNA bases as each array has three to six ZFMs (Figure 4).

On the other hand, oxidative (active) DNA demethylation at targeted promoters of specific genes has been induced by the ten-eleven-translocases (TET) family members. This task was achieved by coupling these enzymes with specially designed ZFs that target those genes’ promoters. For example, in a study conducted to induce oxidative DNA demethylation on human intracellular adhesion molecule 1 (ICAM-1) gene promoter, a hexameric ZF array was coupled to the TET-2 enzyme and used to target the promoter [131]. This intervention led to demethylation of the ICAM promoter and subsequent reactivation of the expression of the gene.

## 4. Summary of Zinc Metalloproteins in Epigenetics and Their Crosstalk

Zinc metalloproteins involved in epigenetics are summarily classified into five main classes. Class I are the writers or markers: they include zinc metalloenzymes that establish epigenetic marks such as DNA methylation, histone methylation acetylation, and ubiquitination. Examples are DNMTs, HMTs, HATs, and EUBLs. Class II is nicknamed erasers due to their ability to erase or remove epigenetic marks such as histone methylation, acetylation, and ubiquitination. Examples include enzymes such as KDMs, HDACs, and DUBm complexes. Class III is made up of a large family of ZFPs that read epigenetic marks such as DNA methylation and histone modifications and subsequently generate signaling pathways by recruiting an array of proteins involved in the signal transduction process, which ultimately lead to the activation or silencing of genes [39,40,112]. This class is termed readers, and their examples include Kaiso, UHRF1, UHRF2, ZFP57, HP1, BPTF, etc. Class IV is made up of a customized combination of readers coupled with writers or erasers. These assemblies are employed in epigenome editing and are referred to as editors.

**Figure 5:**
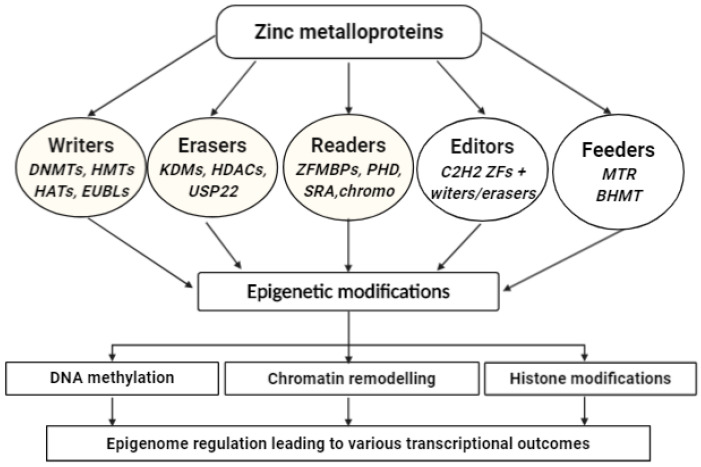
Summary of zinc metalloproteins and their roles in epigenome regulation. Figure 5 summarizes the various classes of zinc metalloproteins that come together to regulate the epigenome. Writers, erasers, readers, editors, and feeders represent the names of the types. These classes of proteins work together in a coordinated manner to regulate the epigenome. Examples of enzymes/proteins in each category are given in abbreviations. BHMT = Betaine homocysteine methyltransferase: a zinc metalloenzyme that catalyzes the formation of methionine from homocysteine and betaine, an essential reaction in DNA and histone methylation; chromo = Chromodomain containing proteins such as heterochromatin protein 1 (HP1), an H3K9 methyl reader that facilitates heterochromatin condensation; C2H2 ZFs = 2-Cysteine-2-Histidine-type of zinc fingers, critical readers of fully methylated DNA; DNMTS = DNA methyltransferases, enzymes that catalyze de novo and maintenance DNA methylation at the CpG islands of fully methylated or hemimethylated DNA; HATs = Histone acetyltransferases, enzymes that catalyze histone lysine acetylation; HDACs = Histone deacetylases, enzymes that catalyze removal acetyl groups from acetyl-lysine residues of histone tails; HMTs = Histone methyltransferases, enzymes that catalyze the methylation of histones on lysine residues; KDMs = Histone lysine demethylases, enzymes that remove methyl groups from the methylated lysine residues of histones; MTR = Methionine synthase, an enzyme that catalyzes the synthesis of methionine from homocysteine and tetrahydrofolate, an essential reaction for DNA and histone acetylation; PHD = Plant homeodomain containing zinc finger proteins, they serve as versatile readers of histone modifications; SRA = SET and RING finger Associated domain-containing proteins: they read hemimethylated DNA. USP22 = Ubiquitin-specific protease 22: the catalytic component of the human deubiquitinating module complex that catalyzes histone deubiquitination. ZF MBPs = Zinc finger methyl binding proteins: readers of fully methylated DNA. Source: Image was created in BioRender (biorender.com (accessed on 23 February 2021)).

A classic example is the hexameric Cys2His2 ZF array associated with the TET-2 enzyme and used for the ICAM gene promoter [128]. Class V are zinc-dependent enzymes nicknamed feeders due to their ability to catalyze critical reactions in the folate-mediated one-carbon metabolism, which is a term used to describe the group of metabolic pathways involved in the supply of one-carbon units such as activated methyl group (in the form of SAM) from folate to DNA, histones, and other biomolecules. In other words, they feed the methylation pathways with active methyl groups for various methylation reactions, including DNA and histone methylations (Figure 5).

Interestingly, readers are involved in crosstalk with writers and erasers, and this interplay regulates the human epigenome, leading to various transcriptional outcomes in both health and disease conditions. For instance, a PHD protein such as UHRF1 can read modified or unmodified histones and then recruit a writer such as DNMT1 to the replication foci. The latter establish epigenetic marks on hemimethylated DNA.

## 5. Conclusions and Future Perspectives

This paper reviewed zinc’s epigenetics role, which depends on zinc metalloproteins’ involvement in epigenome programming. We also discussed here that these proteins contain ZBDs critical for substrate recognition, self-regulation, and catalysis. Most importantly, we demonstrated that an interplay involving the ZBDs of these proteins maintains the highly plastic epigenome in a dynamic state. Therefore, we conclude that zinc is an essential trace metal in epigenetics. However, despite the plethora of ZBD-containing proteins identified and still being discovered, only a few of them have been employed to manipulate the epigenome. Non-communicable chronic diseases such as cancers, diabetes mellitus, and cardiovascular diseases have been associated with aberrant epigenetic changes in the genes related to these diseases; targeting these genes with customized ZBDs could make a remarkable difference in minimizing their burden. For instance, the hypermethylation of oncogenes or demethylation of tumor suppressor genes by epigenetic editors could help control various cancers. Furthermore, studies on transgenerational epigenetic effects at different doses of parental zinc exposure on offspring could unveil how zinc deficiency affects future generations’ health. Thus, the epigenetic burden of diseases could be minimized.

## Figures and Tables

**Table 1:** Summary of zinc-binding domains and their functions.

ZBD	Function	Example(s)	References
ADD	Allosteric control of the catalytic domain	DNMT3a/3b/3L	[31,41,57]
BTB/POZ	Readers of fully methylated DNA (double methylated CpG islands)	Kaiso (ZBTB33), ZBTB4, ZBTB38.	[10,117]
CXXC	Substrate recognition and self-regulation	DNMT1.	[49,50]
JmjC	H3K36me2 demethylation	KDM2B	[82]
KRAB	Reads fully methylated DNA (double methylated CpG islands)	ZFP57	[10]
MYST	HAT activity (H3K9/K14 acetylation)	MOZ, MORF	[94]
PHD	Reads a broad range of histone marks and has E3-ubiquitin ligase activity.	UHRF1, UHRF2, BPTF	[11,40]
RING	E3-ubiquitin ligase	RNF168, PRC1, RNF20-RNF40 complex	[41]
SET	H3K4 methyltransferase activity	SET1A, SET1B	[45,76]
SRA	Reads hemimethylated DNA.	UHRF1, UHRF2.	[121,122]

## Data Availability

Not applicable.
